# Reduced neural specificity for a romantic partner in the nucleus accumbens over relationship duration

**DOI:** 10.1093/scan/nsaf127

**Published:** 2025-12-23

**Authors:** Kenji Fujisaki, Ryuhei Ueda, Ryusuke Nakai, Nobuhito Abe

**Affiliations:** Department of Psychology, Graduate School of Letters, Kyoto University, Kyoto, 606-8501, Japan; Institute for the Future of Human Society, Kyoto University, Kyoto, 606-8501, Japan; Institute for the Future of Human Society, Kyoto University, Kyoto, 606-8501, Japan; Institute for the Future of Human Society, Kyoto University, Kyoto, 606-8501, Japan

**Keywords:** romantic relationships, friends, fMRI, MVPA, nucleus accumbens

## Abstract

Neural processes distinguishing romantic love from opposite-sex friendships remain a key challenge in neuroscience. Research on monogamous prairie voles has revealed that the nucleus accumbens (NAcc) is pivotal for partner-specific processing through plastic changes. However, it remains unclear in humans whether the NAcc differentiates a partner from opposite-sex friends, and how partner-related processing changes as the relationship matures. In a sample of 47 heterosexual male participants, we investigated the neural representations of a female partner, a female friend, and a male friend, in the NAcc, caudate nucleus and putamen. We collected fMRI data from participants during a social incentive delay task designed to elicit neural responses in anticipation of social approval from each of them. Classifier-based multivoxel pattern analysis (MVPA) demonstrated that neural activity patterns in all three regions distinguished the female partner from the female friend. Importantly, similarity-based MVPA revealed that, in the NAcc, the female friend was represented closer to the male friend than to the partner. Furthermore, exploratory analyses indicated that individuals in longer romantic relationships presented less distinguishable neural responses between the partner and the female friend in the NAcc. These findings suggest partner-specific processing in the NAcc, with this specificity diminishing as the relationship matures.

## Introduction

Romantic love is universally observed across societies (Jankowiak and Fischer 1992) and is associated with well-being and health benefits (Lawrence et al. 2019, Hudson et al. 2020). Romantic relationships involve unique cognitions and behaviors, including heightened passion (Hatfield and Sprecher 1986, Sternberg 1986) and intrusive thoughts about one’s partner (Nilakantan et al. 2014, Langeslag and Philippi 2025). Given the ubiquity and distinct characteristics of romantic love, researchers have sought to identify its neurobiological foundations (Bode and Kushnick 2021). Neuroimaging studies have revealed that reward-related regions, including the ventral striatum (nucleus accumbens; NAcc), dorsal striatum (caudate nucleus and putamen), ventral tegmental area (VTA) and ventromedial prefrontal cortex (vmPFC), show increased activity in response to the partner (Bartels and Zeki 2000, Aron et al. 2005, Fisher et al. 2010, Xu et al. 2011, Acevedo et al. 2012).

These regions respond to both primary and secondary rewards, forming interconnected circuits that perform complementary computations to translate cues into motivated behavior (Matyjek et al. 2020, Nguyen et al. 2021). Midbrain dopamine neurons in the VTA broadcast incentive signals to striatal and cortical targets, amplifying the motivational value of reward-predictive cues (‘wanting’) (Robinson and Berridge 1993, Nguyen et al. 2021). The NAcc integrates this dopaminergic input with cortical and limbic information to attribute incentive salience to cues, thereby biasing attention and approach; within the NAcc and the orbitofrontal cortex, small ‘hedonic hotspots’ can modulate the hedonic impact (‘liking’) of outcomes (Kringelbach and Berridge 2017, Nguyen et al. 2021). These mechanisms are expected to motivate partner-directed behaviors (e.g., initiating contact, approaching, and forming and maintaining an intimate relationship) and to shape the hedonic valuation of partner-related outcomes, thereby supporting partner preference.

Consistent with this view, the NAcc appears to play a central role in partner-specific processing. This idea is supported by studies in monogamous prairie voles (*Microtus ochrogaster*), showing that dopamine and oxytocin signaling in the NAcc modulates partner preference formation (Wang et al. 1999, Gingrich et al. 2000, Young et al. 2001, Liu and Wang 2003, Aragona et al. 2003, 2006). Importantly, this effect is not observed in the caudate nucleus or putamen, underscoring the unique role of the NAcc. Moreover, the NAcc exhibits plastic changes as pair bonds mature, which contributes to behaviors that maintain pair bonding (Aragona et al. 2006). These findings suggest that the formation of a distinct representation of the partner in the NAcc is a fundamental mechanism of pair bonds (Walum and Young 2018). Human studies also emphasize the role of the NAcc in partner bonding, demonstrating increased activity in response to oxytocin treatment, which enhances partner preference and avoidance of alternative partners (Scheele et al. 2012, 2013, 2016). Our previous fMRI study employing multivoxel pattern analysis (MVPA) also revealed that the NAcc encodes distinct representations of partners, differentiating them from highly attractive but unfamiliar individuals (Ueda and Abe 2021).

Although these studies highlight the significance of the NAcc in partner bonding, robust evidence for the partner-specific encoding of the NAcc requires identifying differences in its neural responses to partners versus opposite-sex friends, who are often considered potential future partners. However, findings from fMRI studies comparing these two conditions—matched in familiarity and social closeness—have been inconsistent. Some studies reported significantly increased NAcc activity in response to partners compared with opposite-sex friends (Fisher et al. 2010, Acevedo et al. 2012), whereas others reported no significant difference (Bartels and Zeki 2000, Aron et al. 2005), and one study even reported decreased activity (Xu et al. 2011). These divergent findings may stem from the latent romantic interest in opposite-sex friends. From an evolutionary perspective, opposite-sex friends may serve as ‘backup mates’, acting as alternative partners in the event of mate loss or a decline in a current partner’s mate value (Buss et al. 2017, Szymkow and Frankowska 2022). In support of this hypothesis, studies have shown that more than half of extradyadic affairs emerge from close friendships (Labrecque and Whisman 2017).

To fill this research gap, this study aimed to reveal whether opposite-sex friends are represented more similarly to romantic partners or same-sex friends, individuals with a close bond but no romantic interest, by examining spatial patterns of brain activity. We utilized the social incentive delay task (Spreckelmeyer et al. 2009) in combination with two types of MVPA to investigate whether patterns of neural responses differ when male participants anticipate social approval from their female partner, female friend, and male friend—all defined by sex assigned at birth. Building on the prior animal studies (Young et al. 2001, Aragona et al. 2003), we focused our investigation on the NAcc, as well as the caudate nucleus and putamen.

Furthermore, we examined whether the neural specificity of the partner in the NAcc decreases over time. This expectation was informed by previous findings on plastic changes in the NAcc exhibited by mature pair bonds (Aragona et al. 2006) and by evidence in humans that intense passionate love is typically experienced in the early stages of a relationship, tends to decline with neurobiological changes (Marazziti et al. 1999, Marazziti and Canale 2004, Emanuele et al. 2006, Marazziti et al. 2017) and can transition into friendship-like companionate love (García 1998, Ahmetoglu et al. 2010, Langeslag et al. 2013). Given these temporal changes, we expected that the neural specificity of the partner in the NAcc would decrease over time, resulting in increased similarity to the female friend.

## Methods

### Participants

A total of 47 participants whose sex assigned at birth was male were recruited for the study. The sample size was determined based on an a priori power analysis using G*Power Version 3.1.9.6 (Faul et al. 2007). The sample size of 47 participants was calculated to achieve an 80% power for detecting differences in neural dissimilarity between conditions using the Wilcoxon signed-rank test for matched pairs, assuming a medium effect size (Cohen’s *d *= 0.5) and a Bonferroni-corrected significance level of 0.05/3. Inclusion criteria were: (i) age 20–29 years to control for potential age-related effects on neural responses and to align with prior work (Ueda and Abe 2021), (ii) in a romantic relationship with a female partner (sex at birth) for at least three months at the time of the experiment to ensure participants were in committed romantic relationships beyond casual dating, (iii) unmarried and without children to exclude potential confounding effects of marital and parental status, (iv) right-handed, (v) normal or corrected-to-normal vision, and (vi) no history of neurological or psychiatric disorders. Only male participants were included to ensure consistency with prior research (Ueda and Abe 2021) and to avoid potential confounding effects due to sex differences. We confirmed that all participants were heterosexual based on self-reported answers in postexperiment questionnaires and that none of the participants exhibited excessive head motion during the fMRI scanning (ie, repetitive motions larger than 2 mm within each run). Therefore, data from all 47 participants were included in the analysis. The mean age of the participants was 22.3 years (SD = 2.0, median = 22, range = 20–26), and the mean relationship length with their partner was 18.4 months (SD = 16.6, median = 13, range = 3–81). Following a detailed explanation of the study, all participants provided written informed consent in accordance with the guidelines approved by the Kyoto University Ethics Committee. During this process, participants were asked to indicate their sex assigned at birth by choosing one of two options (‘male’ or ‘female’), and all participants identified as male.

### Stimuli

Following previous studies (Kohls et al. 2013, Ueda and Abe 2021), we prepared short video clips and facial images of each participant’s romantic partner (sex at birth: female), female friend (sex at birth: female), and male friend (sex at birth: male), which were used as stimuli during the social incentive delay task. These individuals also provided written informed consent and participated in the recording without the presence of the fMRI participant. We instructed them not to disclose the content of the recording to the fMRI participant until the experiment was completed. For the stimuli used as positive feedback representing social approval (ie, video clips presented following a successful response in the social incentive delay task), we recorded six short video clips of each person displaying smiles with positive gestures: closed-mouth smiles while performing (i) no gesture, (ii) a V-sign, or (iii) a fist pump and toothy smiles while (iv) holding both palms beside their face, (v) clapping, or (vi) waving toward the camera. Each person was guided to make facial expressions and gestures imitating the sample images. For nonapproval stimuli presented following error responses in the social incentive delay task, we recorded a short clip of each person displaying a neutral facial expression with no gestures. We also cropped the neutral facial image from this video clip and used it as a cue stimulus in the task. We edited the recorded video clips and images using Adobe Premiere Pro and Photoshop.

To reduce variability in the nature of the participants’ relationships with their friends, we instructed the participants to select the closest friends who could participate in the study. The mean friendship lengths, defined as the number of months since the first meeting, were 31.7 months (SD = 23.4, median = 27) for female friends and 35.9 months (SD = 25.8, median = 31) for male friends. The mean numbers of days the participants interacted with their partner, the female friend, and the male friend on social media or by phone during the 30 days preceding the experiment were 27.8 (SD = 4.4) 4.3 (SD = 5.0), and 9.8 (SD = 7.6), respectively.

### Social incentive delay task

**Figure 1.**
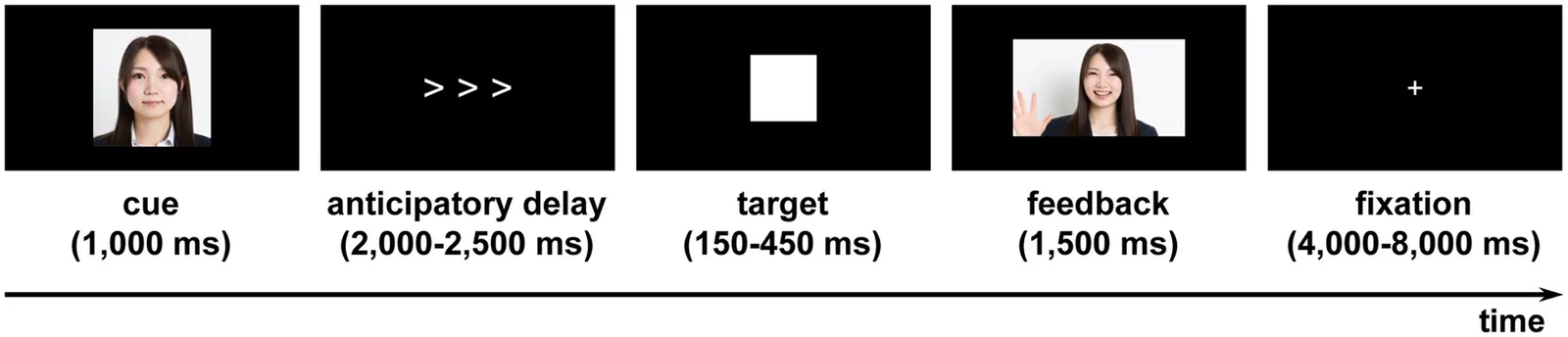
Schematic of the social incentive delay task. Each trial started with a cue stimulus indicating the condition (partner, female friend, or male friend), followed by a variable anticipatory delay phase. A white square target then appeared for a variable duration, during which participants were required to respond quickly by pressing a button. Successful responses resulted in a video clip of the cued person smiling and making a positive gesture, whereas unsuccessful responses resulted in a video clip showing the person making a neutral facial expression.

The participants completed the social incentive delay task (Spreckelmeyer et al. 2009)—a modified version of the monetary incentive delay task (Knutson et al. 2000)—during fMRI scanning. The paradigm is widely used to examine the neural processing of social rewards (Cromwell et al. 2020), in which participants are required to respond quickly to a target stimulus to gain positive feed-back signaling social approval. A brief delay prior to the participant’s attempt to gain social approval minimizes the influence of facial recognition processes on neural activity and reduces confounding effects associated with deliberate evaluation. The protocol was implemented using the Presentation software package (Version 23.1; Neurobehavioral Systems, Inc., Berkeley, CA, www.neurobs.com). A schematic diagram of the task is shown in Fig. 1.

In the present study, the SID task had three conditions: partner, female friend, and male friend. The task consisted of six runs of 18 trials (three conditions × six trials) in a pseudorandomized order with the constraint that the same condition did not appear more than twice consecutively. Each trial started with the presentation of a cue for 1000 ms, followed by an object indicating that the target stimulus would be presented after a variable interval (anticipatory delay phase; 2000–2500 ms). A white square target then appeared for a variable duration (150–450 ms). The participants were instructed to respond by pressing a button with their right index finger as rapidly as possible upon the appearance of the target stimulus. In the subsequent outcome phase, a successful response (ie, hit response, a response made during the presentation of the target stimulus) resulted in the presentation of a short video clip of the cued person (1,500 ms) showing a smile and making a positive nonverbal gesture. To maximize participant engagement, six video clips for each person were presented only once in a pseudorandom order in each run. A miss response (ie, a response made after the target presentation) or an error response (ie, a response made before the target presentation or no response) resulted in the presentation of a short video clip of the cued person with a neutral facial expression without any nonverbal gestures (1500 ms). Unlike in the hit trials, only a single video clip was used per condition in these trials. Following each trial, a fixation cross was presented for a variable intertrial interval (4000–8000 ms). To standardize task difficulty across participants, we applied an adaptive algorithm that dynamically adjusted the target duration based on individual performance, ensuring a consistent hit rate of approximately 66% (Kohls et al. 2013, Abe and Greene 2014, Ueda and Abe 2021). The duration was adjusted in 25-ms increments: shortened if the hit rate exceeded 66% or extended if it fell below 66%. For the first trial of the initial run, the duration was fixed at 300 ms. In the subsequent runs, the duration for the first trial was carried over from the last trial of the previous run. The mean hit response ratio across participants was 60.3% (SD = 2.6%). No participants exhibited a hit rate lower than the mean minus three standard deviations across participants (52.6%), confirming that all participants were seriously engaged in the task.

### Postscan rating tasks and questionnaires

After completing the social incentive delay task, the participants engaged in rating tasks outside the scanner. We used PsychoPy Version 2022.2.4 (Peirce et al. 2019) for stimulus presentation and response recording. They first rated the likeability of the 18 video clips featuring their own partner, female friend, and male friend that were used as positive feedback stimuli in the social incentive delay task. Each video was presented for 1500 ms in a pseudorandom order, followed by a 1000-ms fixation cross. For each clip, participants answered the question, ‘To what extent did you find this video clip likable?’ on a 7-point Likert scale (1 = not at all, 7 = extremely). Ratings were *z* scored for each participant. After the likeability ratings, the participants rated the physical and romantic attractiveness of the same three individuals using the following items: (i) ‘To what extent do you feel physical attraction toward this person?’ and (ii) ‘To what extent do you feel romantic attraction toward this person?’ Both items were answered on a 7-point Likert scale (1 = not at all, 7 = extremely). These ratings were conducted separately, using facial images with neutral expressions as stimuli. The images, which were also used as cue stimuli in the social incentive delay task, were presented on a screen with no time limit.

Following the rating tasks, the participants completed questionnaires. These included the Japanese version of the Triangular Love Scale (Sternberg 1986, Kanemasa and Daibo 2003), the Consensual Non-Monogamy Attitude Scale (Cohen and Wilson 2017), social media interactions, relationship length with the partner, friendship length with the female friend and male friend, a screening item assessing romantic attraction toward males (‘Are males potential romantic partners for you?’; Yes/No) to confirm heterosexual orientation, marital status, and parental status. The Triangular Love Scale was used to assess the three components of love—intimacy, passion, and commitment—through its respective subscales, and the Consensual Non-Monogamy Attitude Scale was used to measure attitudes toward romantic relationships with either a single exclusive partner or multiple partners, respectively. Detailed descriptions and descriptive statistics are provided in the Supplementary Material.

### fMRI data acquisition

Scanning was performed using a 3.0 T MAGNETOM Verio MRI scanner (Siemens, Erlangen, Germany) with a 32-channel head coil. The participants lay in a supine position, and their head motion was restricted with foam padding within the head coil. To ensure optimal vision, participants with inadequate unaided vision were provided with MRI-compatible glasses. Visual stimuli were projected onto a screen and presented to participants via a mirror mounted on the head coil. Button presses were recorded using a fiber-optic button box. Functional imaging was conducted using a T2*-weighted echo–planar imaging (EPI) sequence sensitive to blood-oxygen-level-dependent (BOLD) contrast, with accelerated multiband acquisition, which enabled whole-brain coverage at higher temporal resolution while maintaining signal quality (Feinberg et al. 2010, Moeller et al. 2010, Xu et al. 2013). The acquisition parameters were as follows: repetition time (TR) = 2,000 ms, echo time (TE) = 43 ms, flip angle = 80°, acquisition matrix = 96 × 96, field of view (FOV) = 192 mm, in-plane resolution = 2 × 2 mm, number of axial slices = 76, slice thickness = 2.0 mm with no interslice gap (interleaved acquisition), and multiband acceleration factor = 4. The first five volumes of the 108 samples collected in each run were discarded from analysis because of the nonequilibrium state of magnetization. A high-resolution structural image was also obtained using a T1-weighted magnetization-prepared rapid-acquisition gradient-echo (MPRAGE) pulse sequence, with a spatial resolution of 1 × 1 × 1 mm.

### fMRI data preprocessing and general linear model (GLM)

Image preprocessing and statistical analyses were performed using the Statistical Parametric Mapping software package (SPM12; Wellcome Department of Cognitive Neurology, Institute of Neurology, London, England). The functional data were slice-time corrected with the middle slice acquisition timing (950 ms) as a reference and then realigned to correct for head movements between scans. Each participant’s T1-weighted structural image was coregistered to the mean functional image generated during realignment. The coregistered T1 image was then spatially normalized to a standardized template from the Montreal Neurological Institute (MNI) using unified segmentation (Ashburner and Friston 2005). The realigned functional images were also spatially normalized with the parameters used in normalization of the structural image and resampled to 2 mm isotropic voxels. These normalized, but unsmoothed, functional images were used for the statistical analysis.

A general linear model (GLM) was designed for each participant to model the BOLD signal of each voxel, with regressors created separately for each run. For each run, the GLM contained three regressors representing the anticipatory delay phases for the three conditions (partner, female friend, and male friend) and regressors for the outcome phase of hit and miss responses for each condition. The GLM also incorporated regressors for error responses corresponding to the delay and outcome phases. All the regressors were convolved with a canonical hemodynamic response function. Furthermore, six motion-correction parameters for each run and six run constants were included as regressors of no interest. A high-pass filter (1/128 Hz) was applied to remove low-frequency noise, and a first-order autoregressive model was used to correct for temporal autocorrelation. Beta values corresponding to each condition across runs were estimated for all brain voxels and subsequently converted to *t* values. We used *t* values in the MVPA, as it has been shown that using *t* values improves MVPA performance by reducing the influence of noisy voxels (Misaki et al. 2010).

### ROI definition

To focus on brain regions involved in social reward anticipation, we set the striatal regions, including the NAcc, caudate nucleus, and putamen, as regions of interest (ROIs). These regions have been reported to be activated during the anticipation of social rewards in a meta-analysis of fMRI studies using the social incentive delay task (Martins et al. 2021). We anatomically defined ROI masks using the automated anatomical atlas 3 (AAL3) (Rolls et al. 2020). Because some voxels, particularly those in the ventral area of the NAcc, had missing signals, we refined the masks to include only voxels containing data for at least 80% of the participants (Fig. S1 and Table S1).

### Data analysis

Statistical analyses were conducted using R Version 4.5.0 (R Core Team 2025) and RStudio Version 2024.12.1 + 563 (RStudio Team 2025). All tests were two-tailed, except for the test of classification performance, in which one-tailed tests were used. The Wilcoxon signed-rank tests were conducted using the *exactRankTests* R package Version 0.8.35 and the *coin* R package Version 1.4.3 (Hothorn and Hornik 2022). MVPA was conducted using The Decoding Toolbox Version 3.999F (Hebart et al. 2014).

### Multivoxel pattern analysis

To investigate whether patterns of neural responses differ when participants anticipate social approval from their partner, female friend, and male friend, we conducted two kinds of MVPA: classifier- and similarity-based MVPA (Cohen et al. 2017). Classifier-based MVPA uses machine learning algorithms to examine whether distinguishable patterns of neural activity are observed between stimuli or experimental conditions. In contrast, similarity-based MVPA directly quantifies the degree of similarity in neural activity patterns. Similarity-based MVPA is particularly well suited for the current investigation, as it facilitates direct quantification of neural similarity among the partner, female friend, and male friend conditions, thereby shedding light on the representational positioning of female friend within the higher-order space relative to the partner and male friend in male participants.

#### Classifier-based MVPA

We conducted separate classifier-based MVPA to examine whether neural activity patterns during the anticipation of positive feedback were different between the partner, the female friend, and the male friend in male participants. We used voxelwise BOLD signal patterns as inputs, which were extracted from 18 *t*-maps (ie, 3 conditions × 6 runs) for each participant corresponding to the anticipatory delay phase for each condition in each run. To account for condition-nonspecific response components, the *t* values for each voxel were demeaned by subtracting the mean value across the *t* maps (Op de Beeck 2010, Walther et al. 2016). Within each ROI, classification performances between conditions were computed using a linear support vector machine with a cost parameter (C) of 1 and leave-one-run-out cross-validation. Performance was assessed using the area under the curve (AUC) and averaged across runs. We confirmed that the patterns of activity in the left and right hemispheric ROIs were virtually identical, and the interaction effect between the laterality (left and right ROIs) and the conditions was not significant (Table S2); therefore, classification performances were averaged across both hemispheres for each participant and entered into statistical analysis. To test whether the classification performance in distinguishing between conditions for each ROI was significantly above the chance level (AUC = 0.50, reflecting the expected outcome of random predictions in a binary classification task), we conducted one-sample Wilcoxon signed-rank tests for each pairing (ie, Partner–Female Friend, Partner–Male Friend, and Female Friend–Male Friend) with Bonferroni correction (adjusted *p *=* p *× 3).

#### Similarity-based MVPA

We performed similarity-based MVPA (Kriegeskorte et al. 2008) within each ROI to quantify neural dissimilarity between the three conditions. As in the classifier-based MVPA, we extracted neural activity patterns for each condition in each run from 18 *t*-maps for each ROI and demeaned the *t* values for each voxel. Pairwise Spearman’s rank correlations were then computed between all vectors, resulting in an 18 × 18 representational similarity matrix for each participant. The use of rank correlation allowed us to calculate the similarity without assuming a normal distribution of the *t* values across voxels. Considering confounding within- and between-run correlations (Dimsdale-Zucker et al. 2018), we extracted only between-run correlations from the upper triangular matrix and averaged them for each pairing. We then converted them to dissimilarity indices (1−Spearman’s *ρ*). As with the classifier-based MVPA, we found no significant interaction effect between laterality (left and right ROIs) and conditions (Table S2), and aggregated dissimilarities across hemispheres were used for statistical tests. To investigate whether the dissimilarities differed between pairs of conditions, we conducted Wilcoxon signed-rank tests for matched pairs on the dissimilarities for each comparison (e.g., Partner–Female Friend vs. Female Friend–Male Friend) with Bonferroni correction (adjusted *P *=* P *× 3).

## Results

### Behavioral data

**Figure 2.**
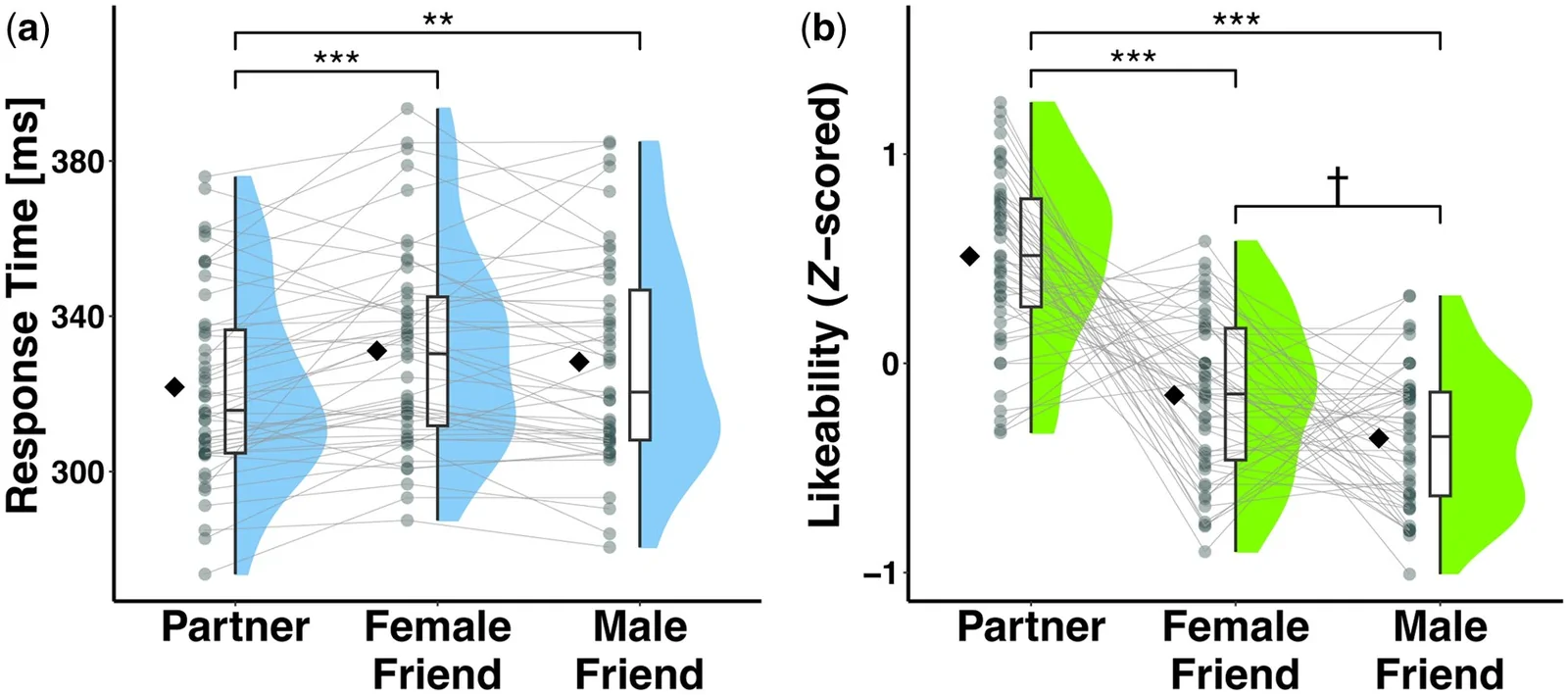
Response times and likeability ratings for each condition. (a) Participants exhibited significantly faster response times in the partner condition than in both the female friend and the male friend conditions. (b) Participants reported significantly greater likeability on video clips in the partner condition than in both the female friend and the male friend conditions. In each panel of behavioral data, half-violin plots represent the data distribution density, and boxplots display the interquartile ranges (IQRs) with median lines. Whiskers extend to the largest value within 1.5 × IQR from the first and third quartiles, and black diamonds represent mean values. Gray lines connect individual data (N = 47) across different condition pairings. ^†^*P *< 0.10, *P < 0.05, ^**^P < 0.01, ^***^P < 0.001, Bonferroni corrected.

We first compared response times between conditions in the social incentive delay task using Wilcoxon signed-rank tests with Bonferroni correction (adjusted *P *=* P *× 3). The participants responded significantly faster in the partner condition than in both the female friend (*W *= 204, adjusted *P *< .001, 95% CI for the median difference = [−12.3, −4.6], effect size *r *= −0.56) and the male friend conditions (*W *= 277, adjusted *P *= .006, 95% CI = [−9.9, −1.9], *r *= −0.44). No significant difference was found between the female friend and the male friend conditions (*W *= 715, adjusted *P *= 0.335, 95% CI = [−0.4, 6.8], *r *= 0.23) (Fig. 2a). These results align with previous findings showing that anticipation of social approval from a partner leads to faster responses (Ueda and Abe 2021), suggesting heightened motivation to obtain positive feedback from partners.

We then conducted the same analysis on likeability ratings. The participants rated the video clips of their partner as more likeable than those of the female friend (*W *= 969, adjusted *P *< 0.001, 95% CI = [0.46, 0.93], *r *= 0.68) and the male friend (*W *= 967, adjusted *P *< 0.001, 95% CI = [0.76, 1.15], *r *= 0.80). There was a trend toward higher ratings for the female friend than the male friend (*W *= 555, adjusted *P *= 0.061, 95% CI = [0.03, 0.49], *r *= 0.34), although this difference did not reach statistical significance (Fig. 2b). Similarly, participants demonstrated a clear preference for their partner in terms of physical attractiveness and romantic attractiveness (Fig. S2).

### Distinguishing neural representations of the partner, female friend, and male friend

**Figure 3.**
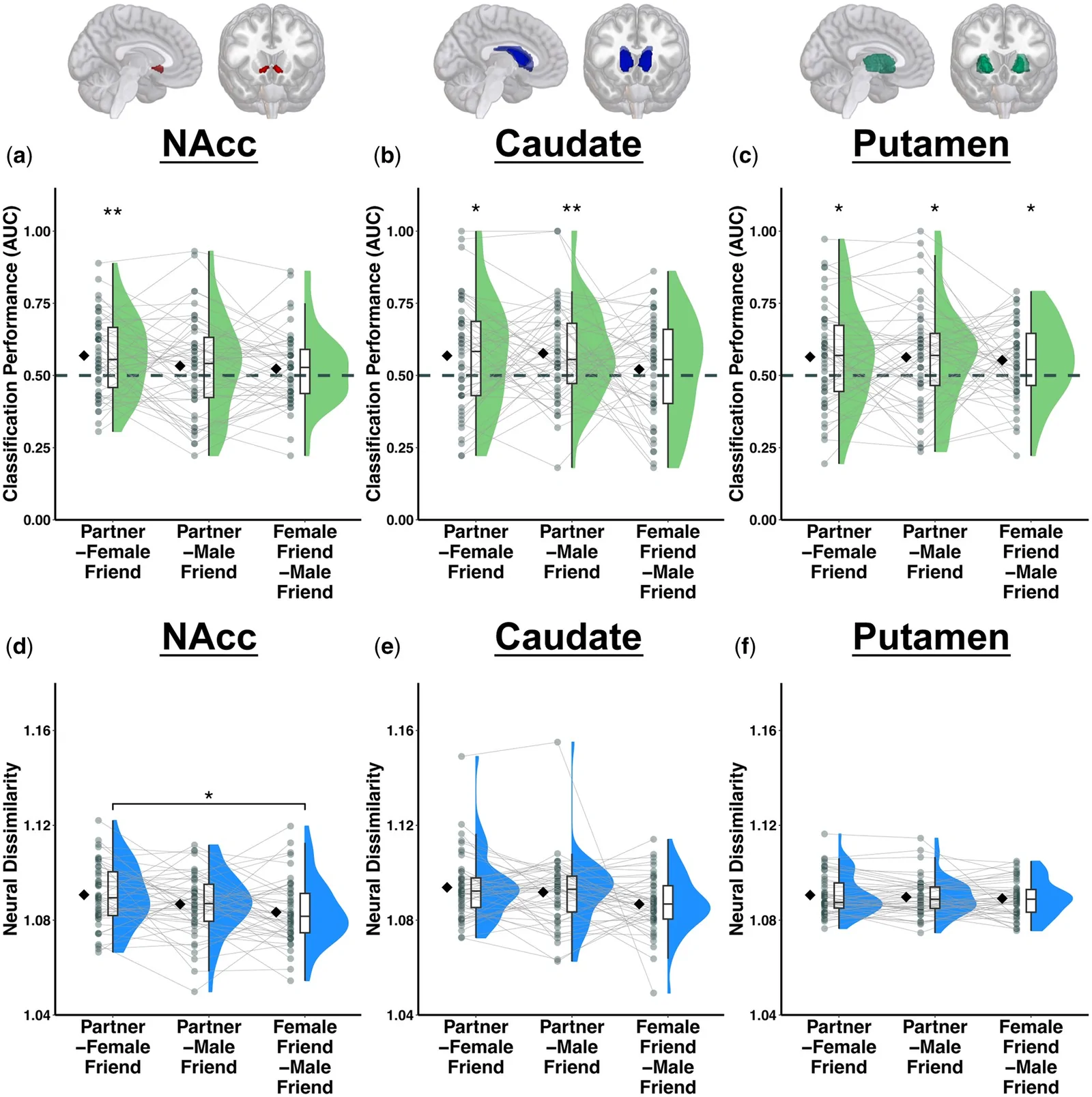
Classification performance and neural dissimilarity for each condition pairing and each ROI. (a)–(c) Classification performance for Partner–Female Friend was consistently significantly above chance level (indicated by the dotted line) across regions. (d)–(f) Only in the nucleus accumbens (NAcc) was the neural dissimilarity for Partner–Female Friend statistically greater than that for the Female Friend–Male Friend. Pairings such as Partner–Female Friend, Partner–Male Friend, and Female Friend–Male Friend represent the combinations of conditions used to calculate classification performance or neural pattern dissimilarity. For example, Partner–Female Friend indicates the classification performance or dissimilarity calculated between the partner and female friend conditions. In each panel, half-violin plots represent the data distribution density, and boxplots display the interquartile ranges (IQRs) with median lines. Whiskers extend to the largest value within 1.5 × IQR from the first and third quartiles, and black diamonds represent mean values. Gray lines connect individual data (N = 47) across different condition pairings. *P < 0.05, **P < 0.01, Bonferroni corrected.

The classifier-based MVPA revealed that neural representations of the partner and the female friend were distinguishable in all ROIs (Table 1, Fig. 3a–c). In the NAcc, the classification performance was significantly above the chance level for the Partner–Female Friend pairing (adjusted *P *= 0.003, *r *= 0.44) but not for the other parings. In the caudate nucleus, the performance was above the chance level for both the Partner–Female Friend (adjusted *P *= 0.037, *r *= 0.33) and the Partner–Male Friend pairings (adjusted *P *= 0.004, *r *= 0.43), whereas it was not significant for the Female Friend–Male Friend pairing. In the putamen, the performance was significantly above the chance level for all parings (Partner–Female Friend: adjusted *P *= 0.035, *r *= 0.33; Partner–Male Friend: adjusted *P *= 0.043, *r *= 0.32; Female Friend–Male Friend: adjusted *P *= 0.015, *r *= 0.37). These results suggest that all the ROIs encoded the committed partner in a different way from the female friend in male participants. We also conducted univariate analyses to compare signal changes across conditions, which revealed no statistically significant differences between the partner and the female friend in the NAcc (the detailed methods and results are provided in the Supplementary Material).

**Table 1.** Descriptive statistics and one-sample Wilcoxon signed-rank test results for classification performance in each ROI.

ROI	Paring	AUC			*W*	Adjusted *P*	95% lower CI	Effect size *r*
		Mean	SD	Median				
NAcc	Partner–Female Friend	0.57	0.14	0.56	850.5	.003^**^	0.53	0.44
	Partner–Male Friend	0.53	0.17	0.54	627.5	.326	0.49	0.18
	Female Friend–Male Friend	0.52	0.13	0.53	636.5	.448	0.49	0.15
Caudate	Partner–Female Friend	0.57	0.19	0.58	744.5	.037^*^	0.52	0.33
	Partner–Male Friend	0.58	0.16	0.56	808	.004^**^	0.53	0.43
	Female Friend–Male Friend	0.52	0.18	0.56	613.5	.645	0.48	0.12
Putamen	Partner–Female Friend	0.56	0.18	0.57	777.5	.035^*^	0.51	0.33
	Partner–Male Friend	0.56	0.18	0.57	739.5	.043^*^	0.51	0.32
	Female Friend–Male Friend	0.55	0.13	0.56	773.5	.015^*^	0.52	0.37

Note: ^*^P  <  .05, ^**^P  <  .01.

Given the absence of significant classification for the Partner–Male Friend pairing in the NAcc, we conducted an exploratory analysis to examine whether individual differences in the relationship between these two targets might account for this result. To capture this relational difference, we used the difference in interaction frequency via social media or phone with the partner versus the male friend over the past 30 days. This analysis revealed that the male participants who communicated more frequently with their partner than with their male friend showed higher classification accuracy for this pairing (Spearman’s *ρ* = 0.36, *P *= 0.012).

Although our primary focus was on the NAcc and other striatal regions, we explored other two regions implicated in social valuation and affective salience, which are also functionally connected with the NAcc, forming part of the reward-processing network—the vmPFC and the anterior insula (aINS) (Craig 2009, Hiser and Koenigs 2018). Since these exploratory analyses were not part of our primary hypotheses, we summarized the findings in the Supplementary Material.

### Representational dissimilarity between the partner, female friend, and male friend

To directly test whether the female friend was represented more similarly to the partner or male friend in male participants, we conducted the similarity-based MVPA. The results are summarized in Table 2 and illustrated in Fig. 3d–f. In the NAcc, the neural dissimilarity was significantly greater for the Partner–Female Friend pairing than for the Female Friend–Male Friend pairing (adjusted *P *= .022, effect size *r *= 0.39). However, no significant differences were detected between the Partner–Female Friend and Partner–Male Friend pairings or between the Partner–Male Friend and Female Friend–Male Friend pairings. These findings suggest that the NAcc is particularly sensitive to neural distinctions between the partner and the Female Friend. Unlike the NAcc, this sensitivity was not observed in the caudate nucleus or putamen, where no significant differences were found across all condition pairings.

**Table 2.** Wilcoxon signed-rank test results for matched pairs of neural dissimilarities in each ROI.

ROI	Comparison	*W*	Adjusted *P*	95% CI	Effect size *r*
NAcc	Partner–Female Friend vs. Partner–Male Friend	729	.245	[−0.0006, 0.0084]	0.26
	Partner–Female Friend vs. Female Friend–Male Friend	814	.022^*^	[0.0017, 0.0122]	0.39
	Partner–Male Friend vs. Female Friend–Male Friend	714	.343	[−0.0009, 0.0085]	0.23
Caudate	Partner–Female Friend vs. Partner–Male Friend	609	1.000	[−0.0034, 0.0054]	0.07
	Partner–Female Friend vs. Female Friend–Male Friend	751	.143	[0.0001, 0.0086]	0.29
	Partner–Male Friend vs. Female Friend–Male Friend	702	.440	[−0.0011, 0.0078]	0.21
Putamen	Partner–Female Friend vs. Partner–Male Friend	684	.624	[−0.0007, 0.0027]	0.18
	Partner–Female Friend vs. Female Friend–Male Friend	617	1.000	[−0.0016, 0.0028]	0.08
	Partner–Male Friend vs. Female Friend–Male Friend	553	1.000	[−0.0022, 0.0020]	−0.02

Note: The ‘Comparison’ column indicates which pairs were compared for their neural dissimilarities. For example, ‘Partner–Female Friend vs. Partner–Male Friend’ compares the neural dissimilarity between the partner and female friend to the neural dissimilarity between the partner and male friend. ^*^P < .05.

### Temporal changes in the neural representations of partners

Although both classifier- and similarity-based MVPA revealed that the neural representation of the partner was generally distinct from the female friend in the NAcc, this representation may change over time, considering the temporal dynamics in romantic relationships. To test this possibility, we conducted exploratory correlation analyses between the neural representations related to the partner (ie, classification performance and neural dissimilarity for the Partner–Female Friend pairing) and relationship length with the partner across participants. For each test, we computed a Spearman’s correlation coefficient between these variables and its 95% confidence intervals using a bootstrap method (10 000 resamples) in the *boot* package Version 1.3-31 (Canty and Ripley 2025) implemented in R.

**Figure 4.**
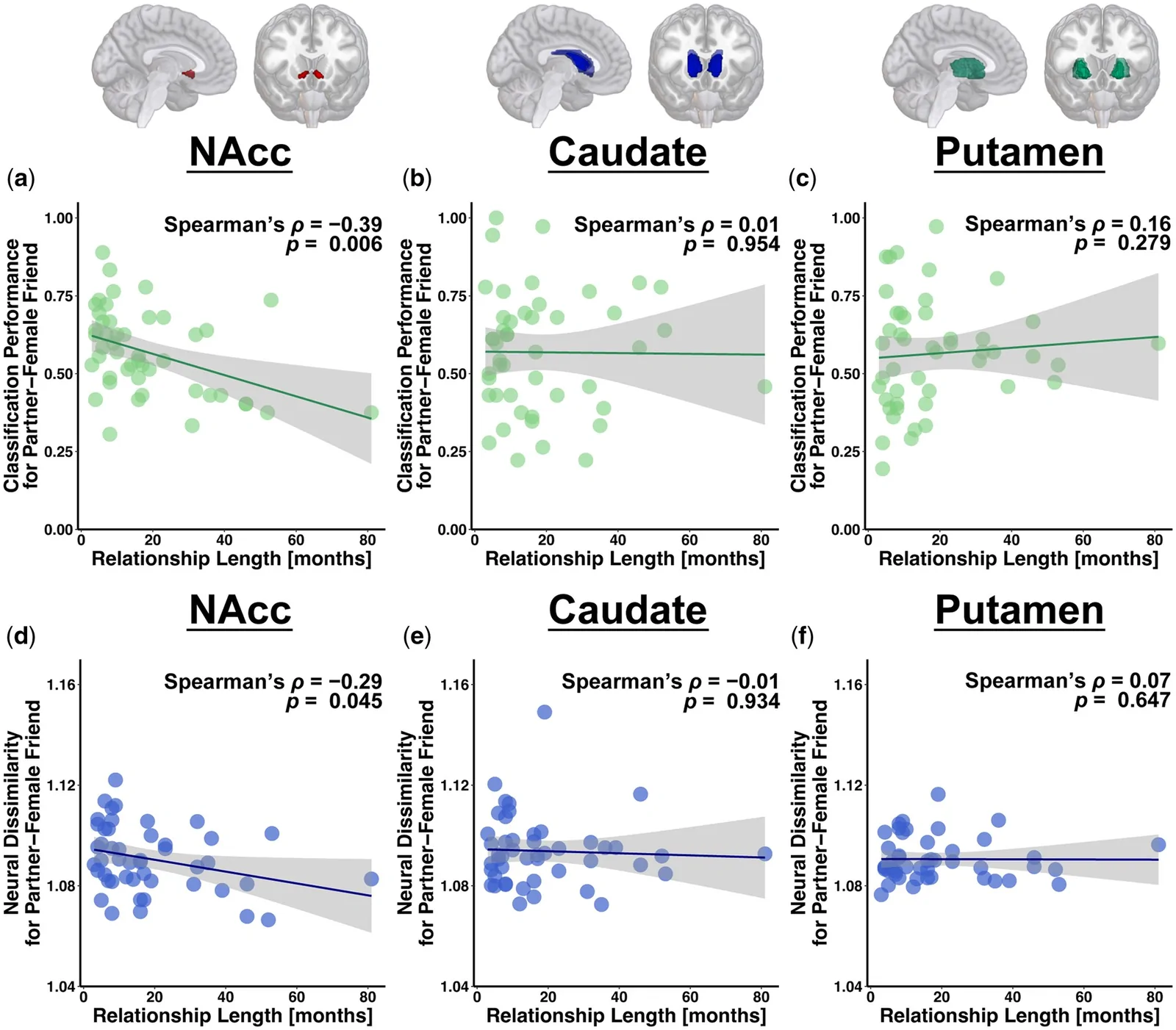
Correlations between relationship length with a current partner and neural dissimilarity between the partner and female friend conditions for each ROI. (a, d) Both classification performance and neural dissimilarity in the nucleus accumbens (NAcc) were significantly negatively correlated with relationship length. Note that we tested the significance based on Spearman’s correlation coefficient, which does not require the assumption of normal distributions of variables. (b, c, e, f) In contrast, no significant correlation was detected for the caudate nucleus or putamen. The solid lines represent regression fits, and the shaded gray areas indicate 95% confidence intervals (N = 47).

As expected, both the classification performance and the neural dissimilarity for the Partner–Female Friend pairing in the NAcc were significantly negatively correlated with relationship length (classification: Spearman’s *ρ* = −0.39, *P *= .006; dissimilarity: Spearman’s *ρ* = −0.29, *P *= .045) (Fig. 4a, d and Table 3), indicating that as the relationship duration increases, the neural differentiation between the partner and the Female Friend in the NAcc decreases. These correlations remained significant even after controlling for the three love components (intimacy, passion, and commitment) assessed by the Triangular Love Scale (classification: partial Spearman’s *ρ* = −0.39, *P *= .010; dissimilarity: partial Spearman’s *ρ* = −0.31, *P *= .040). We also confirmed that the results remained largely unchanged even after excluding one participant identified as an outlier in relationship length (see Supplementary Material). In contrast, no such negative correlations were observed in the caudate nucleus or the putamen (Fig. 4b, c, e, f and Table 3).

**Table 3.** Spearman’s correlation coefficients between relationship length and classification performance and neural dissimilarity for Partner–Female Friend in each ROI.

ROI	Analysis	Correlation type	*ρ*	*P* value	95% CI
NAcc	Classification	Simple	−0.39	.006^**^	[−0.65, −0.09]
		Partial	−0.39	.010^*^	[−0.65, −0.05]
	Dissimilarity	Simple	−0.29	.045^*^	[−0.54, −0.02]
		Partial	−0.31	.040^*^	[−0.56, 0.01]
Caudate	Classification	Simple	0.01	.954	[−0.28, 0.30]
		Partial	0.03	.844	[−0.28, 0.34]
	Dissimilarity	Simple	−0.01	.934	[−0.29, 0.27]
		Partial	0.004	.980	[−0.28, 0.29]
Putamen	Classification	Simple	0.16	.279	[−0.13, 0.42]
		Partial	0.15	.338	[−0.17, 0.41]
	Dissimilarity	Simple	0.07	.647	[−0.24, 0.36]
		Partial	0.05	.746	[−0.25, 0.34]

Note: *P < .05, **P < .01.

## Discussion

We investigated the neural representations of romantic partners, female friends, and male friends during the anticipation of social approval in male participants utilizing both classifier- and similarity-based MVPA in the NAcc, caudate nucleus, and putamen. We found that the neural representation of the partner was significantly different from that of the female friend, particularly in the NAcc. Moreover, we observed that individuals in longer-term relationships tend to exhibit less distinct neural representations of their partners in the NAcc. Our findings suggest that the human NAcc encodes a distinct neural representation of the committed partner, which could be modulated over time.

We emphasize that both types of MVPA consistently demonstrated that the information of partners is specifically represented in the NAcc. Notably, our key findings stem from similarity-based MVPA, revealing that this region, but not the caudate nucleus, or putamen, represents the female friend more similarly to the male friend than to the partner in male participants. These results underscore the partner-specific processing in the NAcc and align with evidence from animal studies on pair bonding. Research on monogamous prairie voles has demonstrated that dopamine and oxytocin signaling within the NAcc, rather than the caudate nucleus and putamen, plays a central role in pair bonding (Young et al. 2001, Aragona et al. 2003, 2006). Moreover, a recent study demonstrated that NAcc dopamine release differentiates a bonded partner from a novel vole when a prairie vole engages in a lever pressing task to gain social access (Pierce et al. 2024). Our social incentive delay task resembles this paradigm, as both tasks require a response to obtain social rewards associated with a partner or alternatives. By extending these observations in prairie voles to humans, our findings highlight the central role of the NAcc in encoding the specificity of the partner. This consistent role of the NAcc across species suggests an evolutionarily conserved mechanism that reinforces bonds with a partner and maintains relationship exclusivity, bridging insights from animal models to human social neuroscience.

Our exploratory correlation analyses revealed that the neural representation of the partner in the NAcc became less distinct from that of the female friend over time in male participants, shedding light on the role of the NAcc in modulating the neural processing of a partner as the relationship matures. These results are consistent with the temporal dynamics of romantic relationships. It is well established that passionate love peaks during the early stage of a relationship and typically diminishes over time, evolving into companionate love, which resembles friendship and is characterized by high levels of intimacy and commitment (García 1998, Ahmetoglu et al. 2010, Langeslag et al. 2013). Our findings on the specificity of the partner in the NAcc as a function of relationship duration likely reflect those psychological fluctuations typically observed in a committed relationship. At the same time, our study raises an intriguing question of how relationships are maintained even as partner-specific representations in the NAcc become less distinct. Insights from prairie vole research may help address this issue, suggesting a neurobiological transition from bond formation to maintenance. During pair-bond formation, activation of D2-like dopamine receptors together with oxytocin receptors in the NAcc facilitates partner preference by linking the neural encoding of partner-related cues with reward (Wang et al. 1999, Gingrich et al. 2000, Young et al. 2001, Liu and Wang 2003, Aragona et al. 2003, 2006). Once the bond is established, two distinct mechanisms are thought to support its maintenance. The first involves an upregulation of D1-like receptors in the NAcc, which promotes selective aggression toward novel potential partners and thereby prevents pair disruption (Aragona et al. 2006, Resendez et al. 2016). The second mechanism involves negative reinforcement, whereby interactions between corticotropin-releasing factor signaling and oxytocin in the NAcc lead individuals to learn that separation from their bonded partner is aversive or stressful (Bosch et al. 2009, Bosch et al. 2016, Walum and Young 2018). Future human research could test whether similar compensatory mechanisms operate in the NAcc as relationships mature, potentially complementing the observed decline in partner specificity. Such investigations would contribute to a better understanding of partner-specific neural dynamics, which are thought to play key roles in relationship formation and maintenance.

Although the male friend would be expected to be distinguished from the partner, the classification accuracy between the partner and the male friend in the NAcc did not reach significance—unlike the Partner–Female Friend classification. Our exploratory analysis showed that this accuracy was positively associated with the difference in interaction frequency between these two targets—a behavioral index of relational closeness. These results suggest that the NAcc, while encoding partner-specific representations, may also encode more general aspects of social bonds, such as closeness or familiarity. This interpretation is consistent with evidence that the NAcc responds to signals from various close individuals, including romantic partners, offspring, parents, siblings, and friends (Feldman 2017, Bortolini et al. 2024, Rinne et al. 2024). Accordingly, the neural distinction between the partner and the friend in the NAcc may become less pronounced when the two relationships are similarly close. While we tried to include the closest male and female friends for each participant, we did not control for perceived familiarity with each of them. Future studies should examine whether the NAcc encodes partner-specific representations beyond the potential effects of this factor.

From a methodological perspective, our analyses using the two types of MVPA provided critical insights into the specific nature of NAcc activity associated with romantic relationships. Although the univariate analysis revealed only a marginal difference between the partner and the female friend in the NAcc (Supplementary Material), the classifier-based MVPA using machine learning algorithms successfully discriminated these conditions. Furthermore, the similarity-based MVPA revealed that the female friend was encoded more similarly to the male friend than to the partner in male participants. These findings address inconsistencies in prior studies relying on traditional univariate analysis, which often struggled to detect reliable differences in NAcc activity between partners and opposite-sex friends (Bartels and Zeki 2000, Aron et al. 2005, Fisher et al. 2010, Xu et al. 2011, Acevedo et al. 2012). By leveraging MVPA, we uncovered the fine-grained sensitivity of the NAcc in distinguishing the female partner from the female friend in male participants.

In the present study, we recruited participants who had been in a romantic relationship for at least three months, focusing on established relationships. Given that romantic love typically emerges during the initiation and early stages of a relationship, accompanied by psychological changes (Vico et al. 2010) and neurobiological alterations (Marazziti et al. 1999, Marazziti and Canale 2004, Emanuele et al. 2006, Marazziti et al. 2017), it is plausible that the NAcc fosters partner-specific representations during these stages. However, how and when such plastic changes occur remain unclear. The partner-specific encoding may develop over the course of several interactions or as early as the initial encounter. Further research is needed to investigate how the neural representation of a partner evolves from the moment of their first meeting.

Although most experimental research on romantic relationships, including the present study, has focused on single time points, our findings highlight the need for future research to investigate how partner-related neural representations change over longer timescales and across different stages of relationships. In long-term relationships, not only duration but also major life transitions—such as cohabitation, marriage or parenthood—can intricately influence the relationship between partners (Gorchoff et al. 2008, Bühler et al. 2021), potentially leading to reorganizations in the neural representation of the partner. In support of this possibility, a study in prairie voles demonstrated that the pregnancy status of the partner altered NAcc dopamine release in males but not in the dorsal striatum or in females (Resendez et al. 2016). Building on these findings, future research should examine whether partner specificity in the NAcc, as demonstrated in the present study with respect to relationship length, can also be modulated across different relationship stages or major life transitions, and whether such adaptive changes in partner specificity follow a nonlinear trajectory—for instance, a U-shaped pattern similar to that observed in marital satisfaction (VanLaningham et al. 2001, Gorchoff et al. 2008, Bühler et al. 2021). Furthermore, it will be informative to compare such trajectories with those observed in other social bonds—such as parental attachment and friendships—to clarify whether partner-related neural changes are unique to romantic relationships or reflect shared neurobiological mechanisms supporting long-term social attachment.

In conclusion, this study is the first to utilize MVPA to investigate neural responses to a romantic partner, a female friend, and a male friend within a single experimental framework in male participants. We found that the human NAcc encodes the specificity of a partner more distinctly during the early stages of a relationship, with this specificity diminishing over time. These results illuminate the role of the NAcc in the formation and maintenance of romantic relationships; however, three limitations warrant consideration. First, our study employed multiband acquisition, which provides several advantages but has also been reported to reduce sensitivity in subcortical regions such as the NAcc (Srirangarajan et al. 2021). Nevertheless, this limitation is more likely to increase the risk of false negatives—that is, failing to detect true effects in the NAcc—rather than false positives and therefore does not undermine the positive results reported here. Second, the analysis examining the association between neural representations in the NAcc and relationship length was exploratory in nature, relying on cross-sectional data with a limited sample size. Future longitudinal research with adequately powered samples could provide deeper insights into how relationship length affects neural representations of the partner within individuals. Third, the current study included only heterosexual male participants, which may limit generalizability. Previous studies have highlighted sex differences in mating strategies (Buss and Schmitt 1993), suggesting that men may be more susceptible to developing romantic interest in nonpartner opposite-sex individuals (Wiederman 1997, Barta and Kiene 2005). Accordingly, the findings of this study may not generalize to individuals of other sexes or sexual orientations. Although a prior fMRI study using univariate analysis has demonstrated broadly similar brain activation maps in response to romantic partners across sexes and sexual orientations (Zeki and Romaya 2010), the small sample size of that study (N = 24) limits the robustness of its conclusions and leaves open the question of whether such consistency extends to multivariate neural representations of partners. Future studies including females and individuals with diverse sexual orientations will be essential for testing the universality of partner-specific neural processing.

## Supplementary Material

nsaf127_Supplementary_Data

## Data and code accessibility

The demographic, behavioral, and fMRI data, as well as the analysis code, are available at: https://doi.org/10.5281/zenodo.14728932.
