# Supplementary material for: Reduced neural specificity for a romantic partner in the nucleus accumbens over relationship duration
Source: Soc Cogn Affect Neurosci. 2025 Dec 23;21(1):nsaf127. doi: 10.1093/scan/nsaf127 (PMC12873753; doi:10.1093/scan/nsaf127)
Supplement: nsaf127_Supplementary_Data [file nsaf127_supplementary_data.docx]

**Supplementary Material**

1. Measures of Romantic Love, Relationship Attitudes, and Social Interactions

The Triangular Love Scale (Sternberg, 1986; Japanese version: Kanemasa and Daibo, 2003), which operationalizes Sternberg’s triangular theory of love into three components—intimacy, passion, and commitment—was used to assess these components toward participants’ current romantic partner. The participants rated each of the 27 items on a 9-point scale, with higher scores indicating stronger levels of each component. The Consensual Non-Monogamy Attitude Scale (Cohen and Wilson, 2017) was employed to assess attitudes toward romantic relationships with either a single exclusive partner or multiple partners. This scale consists of eight items, including three reverse-coded items, with higher scores indicating more positive attitudes toward consensual non-monogamy, which is a relationship orientation that allows multiple romantic or sexual relationships with the agreement of all partners. The Japanese translation of this scale used in this study was developed through collaborative discussions and consensus among three of the authors. Descriptive statistics of the subscales of Triangular Love Scale and the Consensual Non-Monogamy Attitude Scale were as follows: intimacy (mean = 7.83, SD = 1.03, median = 8.20, Cronbach’s *α* = 0.91), passion (mean = 6.76, SD = 1.09, median = 6.80, Cronbach’s *α* = 0.84), commitment (mean = 6.17, SD = 1.62, median = 6.43, Cronbach’s *α* = 0.88), and Consensual Non-Monogamy Attitude Scale (mean = 21.8, SD = 9.57, median = 21.0, Cronbach’s *α* = 0.86).

Regarding the social media interactions, the male participants first reported the numbers of male (mean = 21.3, SD = 14.3) and female (mean = 8.3, SD = 6.8) friends with whom they had communicated via social media or phone in the past 30 days and then reported the numbers of days they had communicated with their partner, female friend and male friend during that time.

**2. ROI-based univariate analysis**

We compared the intensity of neural responses in the anticipatory delay phase across conditions. The normalized functional images used in the MVPA analyses were spatially smoothed with an 4 mm full width, half maximum (FWHM) Gaussian kernel, as Sacchet and Knutson (2013) recommend using smaller kernels (e.g., 4 mm FWHM or less) to minimize spatial displacement in the ventral striatum. Neural responses for each event were estimated by the same GLM as in the MVPA (see Methods in the main text). We used MarsBaR software (Brett et al., 2002) to extract the percentage of the BOLD signal change during the anticipatory delay phase in the ROIs for each condition. We confirmed that the patterns of activity in the left and right hemispheric ROIs were virtually identical and that the interaction effect between laterality (left and right ROIs) and conditions (partner, female friend, and male friend) was not significant (Table S2). Therefore, we used the average signal changes from both hemispheres in the subsequent statistical analyses. We conducted separate Wilcoxon signed-rank tests for each comparison (i.e., Partner vs. Female Friend, Partner vs. Male Friend, and Female Friend vs. Male Friend). Bonferroni correction was applied to *p* values to control for the familywise error rate due to multiple comparisons (adjusted *p* = *p* × 3).

We observed that NAcc activity was greater in the partner condition than in the male friend condition in male participants. However, the difference between the partner and female friend conditions did not reach statistical significance after applying the Bonferroni correction. This finding aligns with previous studies that reported mixed results regarding NAcc activity in response to the partner relative to female friend conditions, including increased activity (Fisher et al., 2010; Acevedo et al., 2012), comparable activity (Bartels and Zeki, 2000; Aron et al., 2005), or decreased activity (Xu et al., 2011). These observations, along with the present results of univariate analysis, underscore the importance of employing advanced analytical approaches, such as MVPA, to uncover subtle neural distinctions. All the results obtained from univariate analysis are shown in Figure S3 and Table S3.

3. Correlation analyses in the NAcc after excluding an outlier

To account for the potential impact of outliers on the correlations between relationship length and neural representations related to the partner (i.e., classification performance or neural dissimilarity for the Partner–Female Friend pairing) in the NAcc, we conducted additional analyses to confirm the robustness of the findings. One participant’s relationship length (81 months) exceeded 3 standard deviations from the mean across participants. Even after excluding this outlier participant, the results of the correlation tests remained largely unchanged: the correlation between relationship length and classification performance remained significant (Spearman’s *ρ* = −0.36, *p* = 0.015, 95% CI = [−0.63, −0.05]), and the correlation with neural dissimilarity remained marginally significant (Spearman’s *ρ* = −0.28, *p* = 0.061, 95% CI = [−0.54, 0.02]). These results were consistent even after controlling for the three love components assessed by the Triangular Love Scale (classification performance: partial Spearman’s *ρ* = −0.34, *p* = 0.025, 95% CI [−0.63, 0.02]; neural dissimilarity: partial Spearman’s *ρ* = −0.27, *p* = 0.084, 95% CI [−0.53, 0.05]). These findings indicate that the observed correlations were not substantially influenced by the presence of the outlier.

**4. Explanatory analyses on the vmPFC and the aINS**

In addition to the striatal regions, we conducted exploratory analyses in the ventromedial prefrontal cortex (vmPFC) and the anterior insula (aINS). Both cortical regions are known to be functionally connected with the striatum, forming part of the reward-processing network (Craig, 2009; Hiser and Koenigs, 2018), and they have also been reported to show greater activity toward the romantic partner (Bartels and Zeki, 2000; Aron et al., 2005; Fisher et al., 2010; Xu et al., 2011; Acevedo et al., 2012). We anatomically defined ROI masks using the automated anatomical atlas 3 (AAL3) (Rolls et al., 2020) for the vmPFC, and the Brainnetome atlas (Fan et al., 2016) for the aINS. Same as the striatal regions, we refined the masks to include only voxels containing data for at least 80% of the participants (Table S1). For each analysis, we confirmed no significant interaction effect between laterality (left and right hemispheres) and conditions (Table S4), and aggregated neural measures across hemispheres were used for statistical tests.

Classifier-based MVPA showed that the partner is distinctively represented in the aINS, but not in the vmPFC (Figure S4 and Table S5). In the vmPFC, classification accuracy was significant for the Female Friend–Male Friend pairing (adjusted *p* = 0.025, *r* = 0.35), whereas it did not exceed chance for the other pairings. Meanwhile, the aINS showed significant above-chance performance for both partner-related pairings (Partner–Female Friend: adjusted *p* < 0.001, *r* = 0.52; Partner–Male Friend: adjusted *p* < 0.001, *r* = 0.53), while it was not significant for the Female Friend–Male Friend pairing. Similarity-based MVPA revealed that while none of the pairwise comparisons reached significance in the vmPFC, the aINS showed a pattern similar to that observed in the NAcc, exhibiting greater neural dissimilarity for the Partner–Female Friend pairing than for the Female Friend–Male Friend pairing (adjusted *p* = 0.044, *r* = 0.35; Figure S4 and Table S6) and no significant differences for other comparisons. We also conducted univariate analyses to compare signal changes across conditions, which revealed no statistically significant differences in the vmPFC, whereas the aINS showed significantly greater signal change for the partner compared with both the female friend and the male friend conditions (Table S7).

In the group-level analysis, the aINS yielded results that were largely comparable to those obtained for the NAcc. Next, we also conducted correlation analyses between the neural representations related to the partner (i.e., classification performance and neural dissimilarity for the Partner– Female Friend pairing) and relationship length with the partner. Importantly, unlike the NAcc (see Figure 4A, D), neither region showed any significant correlations (Figure S5 and Table S8). These results remained unchanged even after controlling for the three love components assessed by the Triangular Love Scale.

These results indicate that the partner was distinguished from the friends in the aINS but not in the vmPFC. The vmPFC is known to be engaged during reward receipt rather than anticipation (Kim et al., 2007; Martins et al., 2021; Ueda and Abe, 2021), which could explain the null findings in the present study. Notably, the aINS showed partner specificity similar to that observed in the NAcc; however, the specificity decreased with relationship duration only in the NAcc, suggesting a functional difference between these regions. The aINS has been implicated in recognizing and differentiating how others relate to oneself and to each other (Lau et al., 2020) and to form part of a circuit in which the NAcc mediates affective signals from the aINS, thereby influencing motivated behavior and reward valuation (Craig, 2009; Clithero et al., 2011). Consistent with these roles, a recent study in monogamous prairie voles suggests that the aINS contributes to the salience detection and affective valuation of social cues, differentiating the partner from an unfamiliar individual, and may act upstream to modulate NAcc function in pair bonding (Vitale et al., 2025). In light of these studies, our findings suggest that the aINS may stably encode partner-related information at the level of emotional salience or social relatedness even as bonds mature, whereas the NAcc may translate such information into motivational signals whose specificity diminishes as relationships mature. Future research should clarify this functional differentiation and how dynamic interactions between these regions support the formation and long-term maintenance of partner bonds.


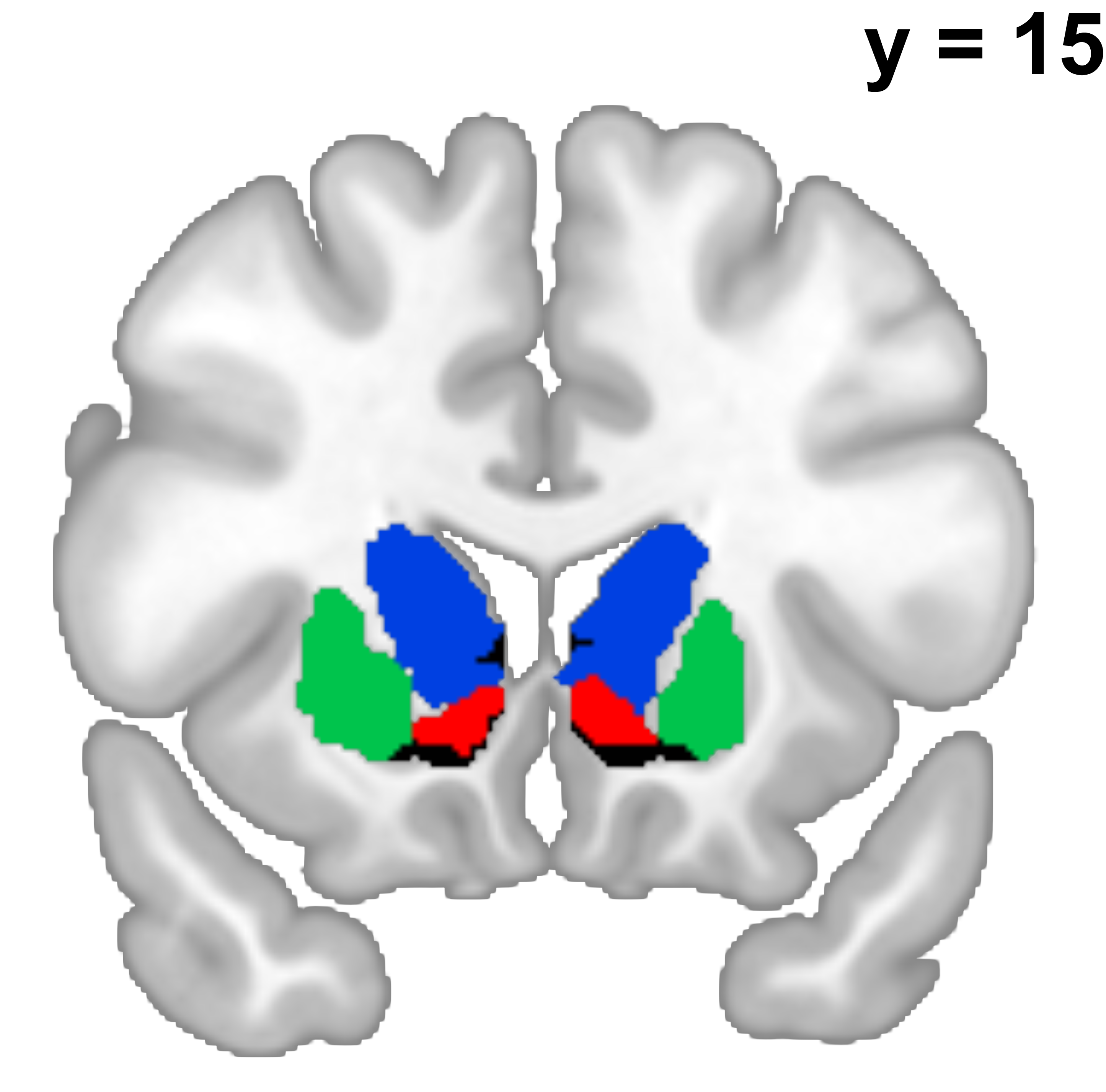


Figure S1. Refined and original ROI masks. The red regions represent the NAcc, the blue regions represent the caudate nucleus, and the green regions represent the putamen in the refined ROI masks. The black areas indicate voxels that were included in the original ROI masks but excluded in the refined ROI masks.

Figure S2. Ratings of physical attractiveness and romantic attractiveness. (A) Participants reported greater ratings of physical attractiveness for their partner than for their friends (partner vs. female friend: *W* = 647.5, adjusted *p* < 0.001, 95% CI for the median difference = [0.50, 2.00], effect size *r* = 0.60; partner vs. male friend: *W* = 690, adjusted *p* < 0.001, 95% CI = [1.00, 2.50], *r* = 0.62). No significant difference was observed between the female friend and the male friend (*W* = 220, adjusted *p* = 0.771, 95% CI = [−0.50, 1.50], *r* = 0.17). The *p* values were Bonferroni corrected for multiple testing. (B) Participants were also generally more romantically attracted to their partner than to their friends (partner vs. female friend: *W* = 833.5, *p* < 0.001, 95% CI = [1.50, 2.50], *r* = 0.77; partner vs. male friend: *W* = 946, adjusted *p* < 0.001, 95% CI = [2.50, 4.00], *r* = 0.84). Furthermore, participants reported greater romantic attractiveness toward their female friend than toward their male friend (*W* = 555.5, adjusted *p* < 0.001, 95% CI = [1.00, 2.50], *r* = 0.65). In each panel, half-violin plots represent the data distribution density, and boxplots display the interquartile ranges (IQRs) with median lines. Whiskers extend to the largest value within 1.5 × IQR from the first and third quartiles, and black diamonds represent mean values. Gray lines connect individual data (N = 47) across different conditions. ^***^*p* < 0.001.

**Figure S3. Signal changes for each condition in the striatal ROIs.** The difference between the partner and female friend conditions was significant in the caudate nucleus and putamen (B, C); however, this difference did not reach significance in the NAcc (A). The mean signal change across the ROIs was greater in the partner condition than in the male friend condition (A–C). In each panel, half-violin plots represent the data distribution density, and boxplots display the interquartile ranges (IQRs) with median lines. Whiskers extend to the largest value within 1.5 × IQR from the first and third quartiles, and black diamonds represent mean values. Gray lines connect individual data (N = 47) across different conditions. ^†^*p* < 0.10, ^**^*p* < 0.01, ^***^*p* < 0.001, Bonferroni corrected.

**Figure S4. Classification performance and neural dissimilarity for each condition pairing in the ventromedial prefrontal cortex (vmPFC) and the anterior insula (aINS).** (A–B) Classification performance for Partner–Female Friend and Partner–Male Friend was significantly above chance level (indicated by the dotted line) in the aINS, but not in the vmPFC. (C–D) In the aINS was the neural dissimilarity for Partner–Female Friend statistically greater than that for the Female Friend–Male Friend. Pairings such as Partner–Female Friend, Partner–Male Friend, and Female Friend– Male Friend represent the combinations of conditions used to calculate classification performance or neural pattern dissimilarity. For example, Partner–Female Friend indicates the classification performance or dissimilarity calculated between the partner and female friend conditions. In each panel, half-violin plots represent the data distribution density, and boxplots display the interquartile ranges (IQRs) with median lines. Whiskers extend to the largest value within 1.5 × IQR from the first and third quartiles, and black diamonds represent mean values. Gray lines connect individual data (N = 47) across different condition pairings. **p* < 0.05, ****p* < 0.001, Bonferroni corrected.

**Figure S5. Correlations between relationship length with a current partner and neural dissimilarity between the partner and female friend conditions for the ventromedial prefrontal cortex (vmPFC) and the anterior insula (aINS).** No significant correlation was detected for both regions. Note that we tested the significance based on Spearman’s correlation coefficient, which does not require the assumption of normal distributions of variables. The solid lines represent regression fits, and the shaded gray areas indicate 95% confidence intervals (N = 47).

Table S1. Voxel counts in the original and refined ROI masks.

| ROI | Hemisphere | Original | Refined |
| --- | --- | --- | --- |
| NAcc | Left | 163 | 81 |
|  | Right | 143 | 71 |
| Caudate | Left | 805 | 794 |
|  | Right | 861 | 839 |
| Putamen | Left | 999 | 978 |
|  | Right | 1062 | 1052 |
| vmPFC | Left | 719 | 362 |
|  | Right | 856 | 529 |
| aINS | Left | 493 | 474 |
|  | Right | 433 | 410 |

**Table S2. Significance of the interaction effect between laterality and conditions across univariate, classification, and pattern dissimilarity measures in each striatal ROI.**

| Variable | ROI | *F* | *Df* | *p* value |
| --- | --- | --- | --- | --- |
| Signal Change | NAcc | 0.738 | (2, 230) | 0.479 |
|  | Caudate | 0.509 | (2, 230) | 0.602 |
|  | Putamen | 0.061 | (2, 230) | 0.941 |
| Classification | NAcc | 0.392 | (2, 230) | 0.676 |
|  | Caudate | 0.052 | (2, 230) | 0.949 |
|  | Putamen | 0.169 | (2, 230) | 0.844 |
| Dissimilarity | NAcc | 0.254 | (2, 230) | 0.776 |
|  | Caudate | 0.143 | (2, 230) | 0.866 |
|  | Putamen | 0.191 | (2, 230) | 0.826 |

Note: The interaction effect was tested using a nonparametric aligned rank transform analysis of variance (ART ANOVA) implemented with the ARTool package (Kay et al., 2021).

Table S3. Wilcoxon signed-rank test results for matched pairs of signal changes across conditions in each striatal ROI.

| ROI | Comparison | *W* | Adjusted *p* | 95% CI | Effect size *r* |
| --- | --- | --- | --- | --- | --- |
| NAcc | Partner vs. Female Friend | 777 | 0.071^†^ | [0.004, 0.048] | 0.33 |
|  | Partner vs.  Male Friend | 853 | 0.005^**^ | [0.008, 0.042] | 0.45 |
|  | Female Friend vs. Male Friend | 613 | 1.000 | [−0.014, 0.024] | 0.08 |
| Caudate | Partner vs. Female Friend | 937 | < 0.001^***^ | [0.019, 0.058] | 0.58 |
|  | Partner vs.  Male Friend | 991 | < 0.001^***^ | [0.028, 0.065] | 0.66 |
|  | Female Friend vs. Male Friend | 668 | 0.829 | [−0.007, 0.022] | 0.16 |
| Putamen | Partner vs. Female Friend | 945 | < 0.001^***^ | [0.023, 0.058] | 0.59 |
|  | Partner vs.  Male Friend | 983 | < 0.001^***^ | [0.024, 0.057] | 0.65 |
|  | Female Friend vs. Male Friend | 553 | 1.000 | [−0.017, 0.016] | −0.02 |

Note: ^†^*p* < 0.10, ***p* < 0.01, ****p* < 0.001.

**Table S4. Significance of the interaction effect between laterality and conditions across univariate, classification, and pattern dissimilarity measures in the vmPFC and the aINS.**

| Variable | ROI | *F* | *Df* | *p* value |
| --- | --- | --- | --- | --- |
| Signal Change | vmPFC | 0.053 | (2, 230) | 0.949 |
|  | aINS | 0.414 | (2, 230) | 0.661 |
| Classification | vmPFC | 1.325 | (2, 230) | 0.268 |
|  | aINS | 0.393 | (2, 230) | 0.676 |
| Dissimilarity | vmPFC | 0.343 | (2, 230) | 0.710 |
|  | aINS | 0.150 | (2, 230) | 0.861 |

Note: The interaction effect was tested using a nonparametric aligned rank transform analysis of variance (ART ANOVA) implemented with the ARTool package (Kay et al., 2021).

**Table S5. Descriptive statistics and one-sample Wilcoxon signed-rank test results for classification performance in the vmPFC and the aINS.**

| ROI | Paring | AUC | | | *W* | Adjusted *p* | 95%  lower CI | Effect size  *r* |
| --- | --- | --- | --- | --- | --- | --- | --- | --- |
|  |  | Mean | SD | Median |  |  |  |  |
| vmPFC | Partner  –Female Friend | 0.51 | 0.15 | 0.50 | 559 | 0.967 | 0.47 | 0.07 |
|  | Partner  – Male Friend | 0.49 | 0.17 | 0.50 | 530 | 1.000 | 0.45 | −0.02 |
|  | Female Friend – Male Friend | 0.55 | 0.16 | 0.57 | 758 | 0.025^*^ | 0.52 | 0.35 |
| aINS | Partner  –Female Friend | 0.62 | 0.20 | 0.61 | 867.5 | < 0.001^***^ | 0.58 | 0.52 |
|  | Partner  – Male Friend | 0.62 | 0.20 | 0.67 | 872.5 | < 0.001^***^ | 0.56 | 0.53 |
|  | Female Friend – Male Friend | 0.52 | 0.16 | 0.51 | 624.5 | 0.545 | 0.48 | 0.13 |

Note: ^*^*p* < 0.05, ^***^*p* < 0.001.

**Table S6. Wilcoxon signed-rank test results for matched pairs of neural dissimilarities in the vmPFC and the aINS.**

| ROI | Comparison | *W* | Adjusted *p* | 95% CI | Effect size *r* |
| --- | --- | --- | --- | --- | --- |
| vmPFC | Partner–Female Friend vs. Partner–Male Friend | 696 | 0.497 | [−0.0015, 0.0075] | 0.20 |
|  | Partner–Female Friend vs. Female Friend–Male Friend | 547 | 1.000 | [−0.0048, 0.0039] | −0.03 |
|  | Partner–Male Friend vs. Female Friend–Male Friend | 386 | 0.180 | [−0.0080, 0.0001] | −0.28 |
| aINS | Partner–Female Friend vs. Partner–Male Friend | 589 | 1.000 | [−0.0039, 0.0046] | 0.04 |
|  | Partner–Female Friend vs. Female Friend–Male Friend | 793 | 0.044^*^ | [0.0014, 0.0120] | 0.35 |
|  | Partner–Male Friend vs. Female Friend–Male Friend | 751 | 0.143 | [0.00004, 0.00123] | 0.29 |

Note: The "Comparison" column indicates which pairs were compared for their neural dissimilarities. For example, “Partner–Female Friend vs. Partner–Male Friend” compares the neural dissimilarity between the partner and female friend to the neural dissimilarity between the partner and male friend. ^*^*p* < 0.05.

Table S7. Wilcoxon signed-rank test results for matched pairs of signal changes across conditions in the vmPFC and the aINS.

| ROI | Comparison | *W* | Adjusted *p* | 95% CI | Effect size *r* |
| --- | --- | --- | --- | --- | --- |
| vmPFC | Partner vs. Female Friend | 635 | 1.000 | [−0.012, 0.026] | 0.11 |
|  | Partner vs.  Male Friend | 693 | 0.527 | [−0.006, 0.033] | 0.20 |
|  | Female Friend vs. Male Friend | 642 | 1.000 | [−0.010, 0.023] | 0.12 |
| aINS | Partner vs. Female Friend | 931 | < 0.001^***^ | [0.029, 0.078] | 0.57 |
|  | Partner vs.  Male Friend | 1022 | < 0.001^***^ | [0.042, 0.085] | 0.71 |
|  | Female Friend vs. Male Friend | 728 | 0.251 | [−0.002, 0.028] | 0.25 |

Note: ****p* < 0.001.

**Table S8. Spearman’s correlation coefficients between relationship length and classification performance and neural dissimilarity for Partner–Female Friend in the vmPFC and the aINS.**

| ROI | Analysis | Correlation Type | *ρ* | *p* value | 95% CI |
| --- | --- | --- | --- | --- | --- |
| vmPFC | Classification | Simple | −0.24 | 0.099 | [−0.50, 0.05] |
|  |  | Partial | −0.18 | 0.234 | [−0.47, 0.13] |
|  | Dissimilarity | Simple | −0.24 | 0.104 | [−0.47, 0.02] |
|  |  | Partial | −0.19 | 0.227 | [−0.43, 0.11] |
| aINS | Classification | Simple | −0.12 | 0.403 | [−0.39, 0.16] |
|  |  | Partial | −0.13 | 0.400 | [−0.39, 0.17] |
|  | Dissimilarity | Simple | −0.06 | 0.666 | [−0.36, 0.24] |
|  |  | Partial | −0.06 | 0.710 | [−0.34, 0.24] |

# Supplementary References

Acevedo B.P., Aron A., Fisher H.E., et al. (2012). Neural correlates of long-term intense romantic love. *Social Cognitive and Affective Neuroscience*, 7, 145–59

Aron A., Fisher H., Mashek D.J., et al. (2005). Reward, Motivation, and Emotion Systems Associated With Early-Stage Intense Romantic Love. *Journal of Neurophysiology*, 94, 327–37

Bartels A., Zeki S. (2000). The neural basis of romantic love: *NeuroReport*, 11, 3829–34

Brett, M., Anton, J.-L., Valabregue, R., et al. (2002). Region of interest analysis using an SPM toolbox [Abstract]. In: 16. Presented at the 8th International Conference on Functional Mapping of the Human Brain. Sendai, Japan: NeuroImage.

Clithero, J.A., Reeck, C., Carter, R.M., et al. (2011). Nucleus Accumbens Mediates Relative Motivation for Rewards in the Absence of Choice. *Frontiers in Human Neuroscience*, 5

Cohen M.T., Wilson K. (2017). Development of the Consensual Non-Monogamy Attitude Scale (CNAS). *Sexuality & Culture*, 21, 1–14

Craig, A.D.B. (2009). How do you feel--now? The anterior insula and human awareness. *Nature Reviews. Neuroscience*, 10, 59–70

Fan, L., Li, H., Zhuo, J., et al. (2016). The Human Brainnetome Atlas: A New Brain Atlas Based on Connectional Architecture. *Cerebral Cortex*, 26, 3508–26

Fisher H.E., Brown L.L., Aron A., et al. (2010). Reward, Addiction, and Emotion Regulation Systems Associated With Rejection in Love. *Journal of Neurophysiology*, 104, 51–60

Hiser, J., Koenigs, M. (2018). The Multifaceted Role of the Ventromedial Prefrontal Cortex in Emotion, Decision Making, Social Cognition, and Psychopathology. *Biological Psychiatry*, 83, 638–47

Kanemasa, Y., Daibo, I. (2003). Three components in the triangular theory of love and intimate opposite-sex relationships. *The Japanese Journal of Research on Emotions*, 10, 11–24

Kay, M., Elkin, L.A., Higgins, J.J., et al. (2021). ARTool: Aligned Rank Transform for Nonparametric Factorial ANOVAs

Kim, H., Adolphs, R., O’Doherty, J.P., et al. (2007). Temporal isolation of neural processes underlying face preference decisions. *Proceedings of the National Academy of Sciences*, 104, 18253–58

Lau, T., Gershman, S.J., Cikara, M. (2020). Social structure learning in human anterior insula. *eLife*, 9, e53162

Martins, D., Rademacher, L., Gabay, A.S., et al. (2021). Mapping social reward and punishment processing in the human brain: A voxel-based meta-analysis of neuroimaging findings using the social incentive delay task. *Neuroscience & Biobehavioral Reviews*, 122, 1–17

Rolls, E.T., Huang, C.-C., Lin, C.-P., et al. (2020). Automated anatomical labelling atlas 3. *NeuroImage*, 206, 116189

Sacchet, M.D., Knutson, B. (2013). Spatial smoothing systematically biases the localization of reward-related brain activity. *NeuroImage*, 66, 270–77

Sternberg, R.J. (1986). A triangular theory of love. *Psychological Review*, 93, 119–35

Ueda R., Abe N. (2021). Neural Representations of the Committed Romantic Partner in the Nucleus Accumbens. *Psychological Science*, 32, 1884–95

Vitale, E.M., Tbaba, A.H., Tam, K., et al. (2025). Opposite‐sex pairing alters social‐induced GCaMP and dopamine activity in the insula of male prairie voles. *Annals of the New York Academy of Sciences*, 1548, 137–47

Xu X., Aron A., Brown L., et al. (2011). Reward and motivation systems: A brain mapping study of early‐stage intense romantic love in Chinese participants. *Human Brain Mapping*, 32, 249–57
